# Molecular cloing and bioinformatics analysis of lactate dehydrogenase from *Taenia multiceps*

**DOI:** 10.1007/s00436-017-5568-x

**Published:** 2017-08-01

**Authors:** Cheng Guo, Yu Wang, Xing Huang, Ning Wang, Ming Yan, Ran He, Xiaobin Gu, Yue Xie, Weimin Lai, Bo Jing, Xuerong Peng, Guangyou Yang

**Affiliations:** 1 Department of Parasitology, College of Veterinary Medicine, Sichuan Agricultural University, Chengdu, 611130 China; 2 Chengdu Agricultural Science and Technology Vocational College, Chengdu, 611130 China; 3 College of Life and Basic Sciences, Sichuan Agricultural University, Chengdu, 611130 China

**Keywords:** *Taenia multiceps*, Lactate dehydrogenase, Bioinformatic analysis, Indirect ELISA

## Abstract

*Coenurus cerebralis*, the larval stage (metacestode or coenurus) of *Taenia multiceps*, parasitizes sheep, goats, and other ruminants and causes coenurosis. In this study, we isolated and characterized complementary DNAs that encode lactate dehydrogenase A (Tm-LDHA) and B (Tm-LDHB) from the transcriptome of *T. multiceps* and expressed recombinant Tm-LDHB (rTm-LDHB) in *Escherichia coli*. Bioinformatic analysis showed that both Tm-LDH genes (LDHA and LDHB) contain a 996-bp open reading frame and encode a protein of 331 amino acids. After determination of the immunogenicity of the recombinant Tm-LDHB, an indirect enzyme-linked immunosorbent assay (ELISA) was developed for preliminary evaluation of the serodiagnostic potential of rTm-LDHB in goats. However, the rTm-LDHB-based indirect ELISA developed here exhibited specificity of only 71.42% (10/14) and sensitivity of 1:3200 in detection of goats infected with *T. multiceps* in the field. This study is the first to describe LDHA and LDHB of *T. multiceps*; meanwhile, our results indicate that rTm-LDHB is not a specific antigen candidate for immunodiagnosis of *T. multiceps* infection in goats.

## Introduction

*Coenurus cerebralis* is the metacestode stage of *Taenia multiceps*, which usually inhabits the central nervous system and subcutaneous and intramuscular tissues of herbivorous mammals, especially sheep, goats, and cattle, and can cause severe coenurosis (Dehghani et al. 2016; Oryan et al., 2015a, b). Coenurosis is distributed extensively (including in Europe, the USA, Africa and Asia) (Huang et al. 2015; Li et al. 2015; Merbl et al. 2014; Varcasia et al. 2015) and leads to huge socioeconomic losses to the livestock breeding industry in many countries. Coenurosis is also a serious zoonosis and can pose threats to the public (Al-Riyami et al. 2015; Nie et al. 2013; Varcasia et al. 2015).

Dehydrogenase enzymes are important catalysts in biological redox reactions, involved in detoxification and various other physiological processes. Lactate dehydrogenase (LDH) plays a central role in regulating glycolysis (Alcazar et al. 2000; Imagawa et al. 2006). The LDH isoenzymes are usually composed of both LDH-A and LDH-B subunits and have different biochemical properties and physiological functions in different tissues (Alcazar et al. 2000). Studies of LDH are useful for examination of the evolution of a species, and in the study of development and growth of parasites and animals (Liwak and Ananvoranich 2009). Gossypol and various antiparasitic drugs including artemisinin and praziquantel can inhibit the activity of the LDHs of *Schistosoma japonicum* (Xiao et al. 1999), *Taenia asiatica* (Chen et al. 2010), *Clonorchis sinensis* (Yang et al. 2006), *Plasmodium falciparum* and *Toxoplasma gondii* (Dando et al. 2001). Thus, LDH is an ideal target for development of antiparasitic drugs.

Given that there is no information on the LDHs of *Taenia multiceps* available to date and the importance of the biochemical and physiological functions of this enzyme in parasites, we isolated and characterized LDHA and LDHB from *T. multiceps* and assessed the immunogenicity of recombinant Tm-LDHB and its serodiagnostic potential in an indirect enzyme-linked immunosorbent assay (ELISA). These results should contribute to new antiparasitic drug development and understanding of the biological functions of LDH in cestodes.

## Materials and methods

### Parasites and serum

Coenuri were obtained from the brains of goats that were naturally infected. All animals were processed in strict accordance with the animal protection law of the People’s Republic of China (release date: September 18, 2009). All serum samples were collected from farms in Sichuan Province, China. Five *T. multiceps-*positive serum samples and seven *Cysticercus tenuicollis-*positive serum samples were obtained from naturally infected goats, while seven *Echinococcus granulosus*-positive serum samples were obtained from naturally infected sheep*.* Twenty-four negative samples were collected from cestode-free goats (confirmed by autopsy). All samples were maintained at − 20 °C until use.

### Cloning, expression, and purification of rTm-LDH

Total RNA was extracted using Trizol reagent (Tiangen, Beijing, China) and then reverse-transcribed into complementary DNA according to the RevertAid First Strand cDNA Synthesis Kit manufacturer’s instructions (MBI Fermentas, Germany). The cDNA sequence of Tm-LDHA was amplified using primers designed from Unigene 18396 of the assembled *T. multiceps* transcriptome dataset, which is homologous to the LDH of *T. asiatica* (GenBank accession no: EF420317.1). The cDNA sequence of Tm-LDHB was amplified using primers designed from Unigene 19054 of the assembled *T. multiceps* transcriptome dataset, which is homologous to the LDH sequence of *T. solium* (GenBank accession no: GU571143.1). The primers for Tm-LDHA were the following: 5′-GAAGTTGTTTGCGGGGAAT-3′ and 5′-CCTCACAATCCACACAGTAATA-3′. The primers for Tm-LDHB were 5′-CGGAATTCATGGCTGAACATTCTATCCTCG-3′ and 5′-CGCTCGAGTCACCATTTGATACCAGAAGTAGTTT-3′, with *Eco*RI and *Xho*I restriction enzyme sites (underlined). After amplification and gel purification, the target Tm-LDHB fragment was integrated into expression vector pET32a(+) (Takara, Dalian, China) and transformed into *Escherichia coli* BL21 (DE3) competent cells. The recombinant protein was expressed on induction by 1 mM isopropyl β-D-1-thiogalactopyranoside (IPTG) and purified using Ni^2+^ affinity chromatography (Bio-Rad, Hercules, CA, USA), following the manufacturer’s instructions. Protein purification was analyzed by 12% sodium dodecyl sulfate polyacrylamide gel electrophoresis (SDS-PAGE).

### Bioinformatic analyses

ORF Finder (http://www.ncbi.nlm.nih.gov/gorf/gorf.html) was used to predict open reading frames; basic physicochemical properties, the estimated half-lives, and stability coefficients of proteins were predicted by ProtParam (http://web.expasy.org/protparam/); signal peptides were predicted by SignalP (http://www.cbs.dtu.dk/Services/SignalP/); transmembrane regions were predicted by the TMHMM2.0 server (http://www.cbs.dtu.dk/services/TMHMM-2.0); subcellular localizations were predicted by BaCelLo (http://gpcr.biocomp.unibo.it/bacello/pred.htm); B-cell epitopes were predicted by the BepiPred 1.0b server (http://www.cbs.dtu.dk/services/BepiPred/); Swiss-model (http://swissmodel.expasy.org/) was used to predict three-dimensional protein structures; and MEGA 5.1 was used for phylogenetic analysis.

### Western blotting

For immunoblotting, the recombinant LDH protein was transferred onto a nitrocellulose (NC) filter membrane after separation by 15% SDS-PAGE. Subsequently, the membranes were blocked with 5% (*w*/*v*) skim milk for 2 h at room temperature, then incubated overnight with *T. multiceps*-positive goat serum (1:200 *v*/*v* dilution) at 4 °C. After five washes with Tris-buffered saline Tween-20 buffer (TBST), 1:1000 diluted horseradish peroxidase (HRP)-conjugated rabbit anti-goat IgG (Bio-Rad) was added and further incubated for 2 h at room temperature. After washing with TBST, the enhanced HRP-DAB chromogenic substrate kit (Tiangen) was used to visualize reactions.

### Development of Tm-LDHB indirect ELISA (iELISA)

Recombinant TmLDH (rTmLDH) in 0.1 mM carbonate buffer (pH 9.6) was diluted to concentrations of 9.6, 4.8, 2.4, 1.2, 0.6, and 0.3, respectively. Ninety-six-well ELISA plates were coated with 100-μL diluted protein overnight at 4 °C as described previously (Crowther 2009). All wells were washed three times with 300 μL PBS buffer containing 0.05% Tween-20 (PBST). Each well was blocked for 2 h at 37 °C with 5% skim milk diluted with PBS. After three washes with PBST, the wells were incubated for 1 h at 37 °C with 100 μL of serum samples with dilutions 1:20, 1:40, 1:80, 1:160, 1:320, and 1:640 in PBS. Following washing steps, 100 μL rabbit anti-goat IgG-HRP conjugate (Boster Bio-project Co., Wuhan, China) was added to each well at a dilution of 1:4000, and the plates were incubated for 1 h at 37 °C. After washing, each well was subsequently incubated with 100 μL 3,3′,5,5′-tetramethylbenzidine (Tiangen) at 37 °C for 15 min; color development reactions were stopped with 100 μL of 2 M H_2_SO_4_. Finally, the optical density (OD) of each well was measured at 450 nm (OD450). Other optimal conditions were explored according to a previous report (Lu et al. 2014). The optimal working conditions were regarded as those that gave the maximum difference in values of OD450 between positive and negative sera. Twenty-four negative serum samples were used to determine the cut-off value, which was calculated as the mean + 3 standard deviations of the OD450 value of the negative serum samples in the optimal working conditions.

### Specificity and sensitivity of the Tm-LDHB iELISA

In the optimum conditions for the iELISA, we assessed potential cross-reactivity with seven *C. tenuicollis*-positive serum samples and seven *E. granulosus*-positive serum samples to determine the specificity of the rTm-LDHB iELISA. Three *T. multiceps*-positive serum samples twofold serially diluted from 1:50 to 1:25600 were used to evaluate the sensitivity of the rTm-LDHB iELISA.

## Results

### Bioinformatics analysis of Tm-LDH

Both *T. multiceps* LDH cDNA sequences (LDHA and LDHB) contained a 996-bp open reading frame and encoded a predicted polypeptide consisting of 331 amino acids. No signal peptides were predicted in Tm-LDH. Tm-LDHB had a predicted molecular weight (MW) of 35.53 kDa and a pI of 7.21, and Tm-LDHA had a predicted MW of 35.29 kDa and a pI of 8.03. The estimated half-lives of Tm-LDHA and Tm-LDHB were 30 h, with stability coefficients of 28.59 and 17.30, respectively, suggesting that Tm-LDHA and Tm-LDHB are stable. No transmembrane regions were predicted in either Tm-LDHA or Tm-LDHB. Subcellular localization analysis indicated that Tm-LDHA located in the cytoplasm while Tm-LDHB located in the mitochondria.

**Fig. 1:**
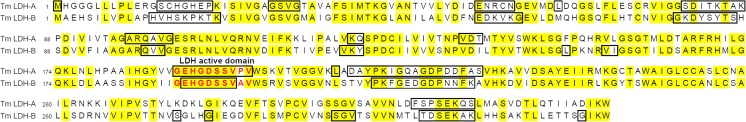
B-cell epitope prediction in *T. multiceps* LDHA and LDHB. LDH active sites are highlighted with *red letters*

**Fig. 2:**
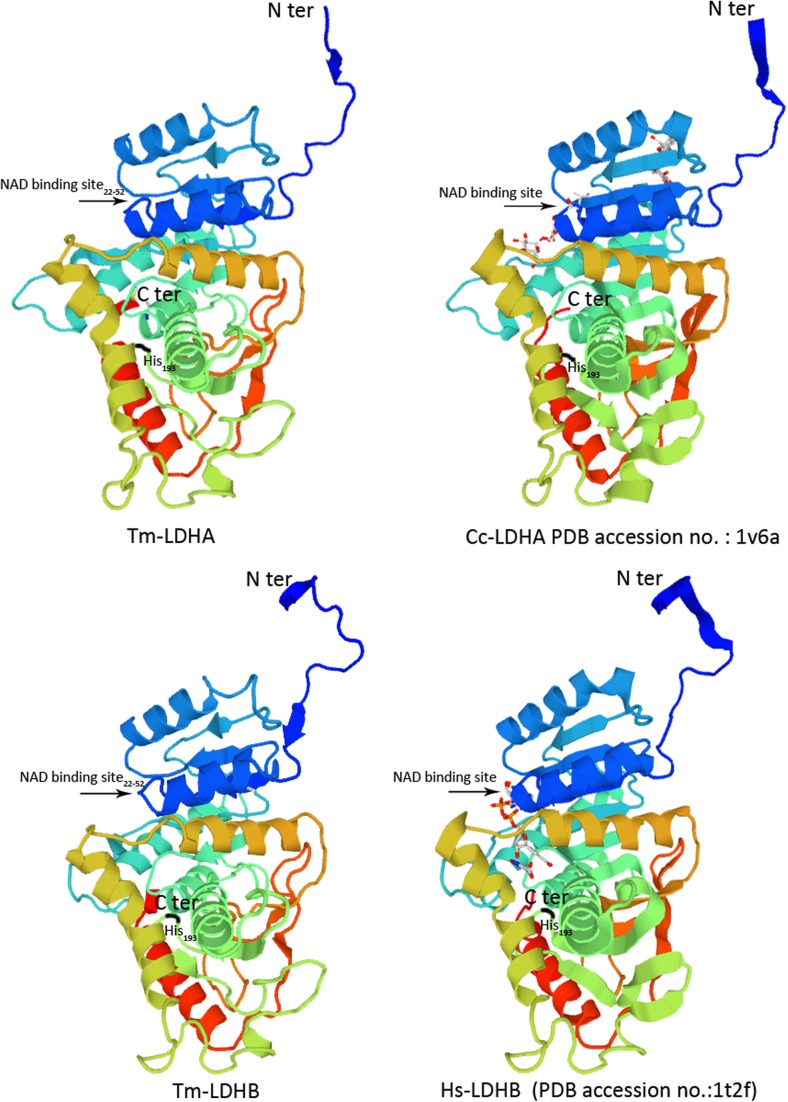
Three-dimensional structure model of *T. multiceps* LDHA and LDHB. The structure of Tm-LDHA was based on the crystal structure of *Cyprinus carpio* LDHA (Cc-LDHA; PDB accession code 1V6A). The structure of Tm-LDHB was based on the crystal structure of *Homo sapiens* LDH-B (Hs-LDHB; PDB accession code 1T2F)

Thirteen B-cell epitopes (amino acids (aa) 1, 14–20, 28–31, 54–58, 64, 80–87, 97–102, 125–126, 139–141, 190–199, 210, 212–228, and 307–314) were found in Tm-LDHA, and fourteen (aa 13–21, 54–59, 79–86, 99–101, 124–126, 152, 157–158, 191–197, 215–227, 277, 281, 297–299, 308–314, 328) were predicted in Tm-LDHB (shown in black boxes in Fig. 1). Furthermore, Tm-LDHA has eight predicted N-myristoylation sites, five protein kinase C phosphorylation sites, three casein kinase II phosphorylation sites, one NAD binding site, and one tyrosine kinase phosphorylation site. Tm-LDHB has five predicted protein kinase C phosphorylation sites, five casein kinase II phosphorylation sites, four N-glycosylation sites, four N-myristoylation sites, one NAD binding site, and one tyrosine kinase phosphorylation site. Notably, Tm-LDHA and Tm-DLHB appeared to share a similar LDH active site (GEHGDS) (Fig. 1). Sequence analysis revealed that this LDH active site is conserved in *T*. *multiceps* and other cestode species. In Tm-LDHA (aa 190–199) and Tm-LDHB (aa 191–197), this active site overlapped with a B-cell epitope (Fig. 1). Secondary structure analysis predicted that Tm-LDHA contains 31.42, 27.49, and 28.7% alpha helix, β-strand, and loop, respectively. A similar structure composition was found for Tm-LDHB, with the corresponding values 33.53, 30.51, and 25.98%. In addition, the three-dimensional structures of Tm-LDHA and Tm-LDHB were modeled (Fig. 2).

### Phylogenetic analysis

**Fig. 3:**
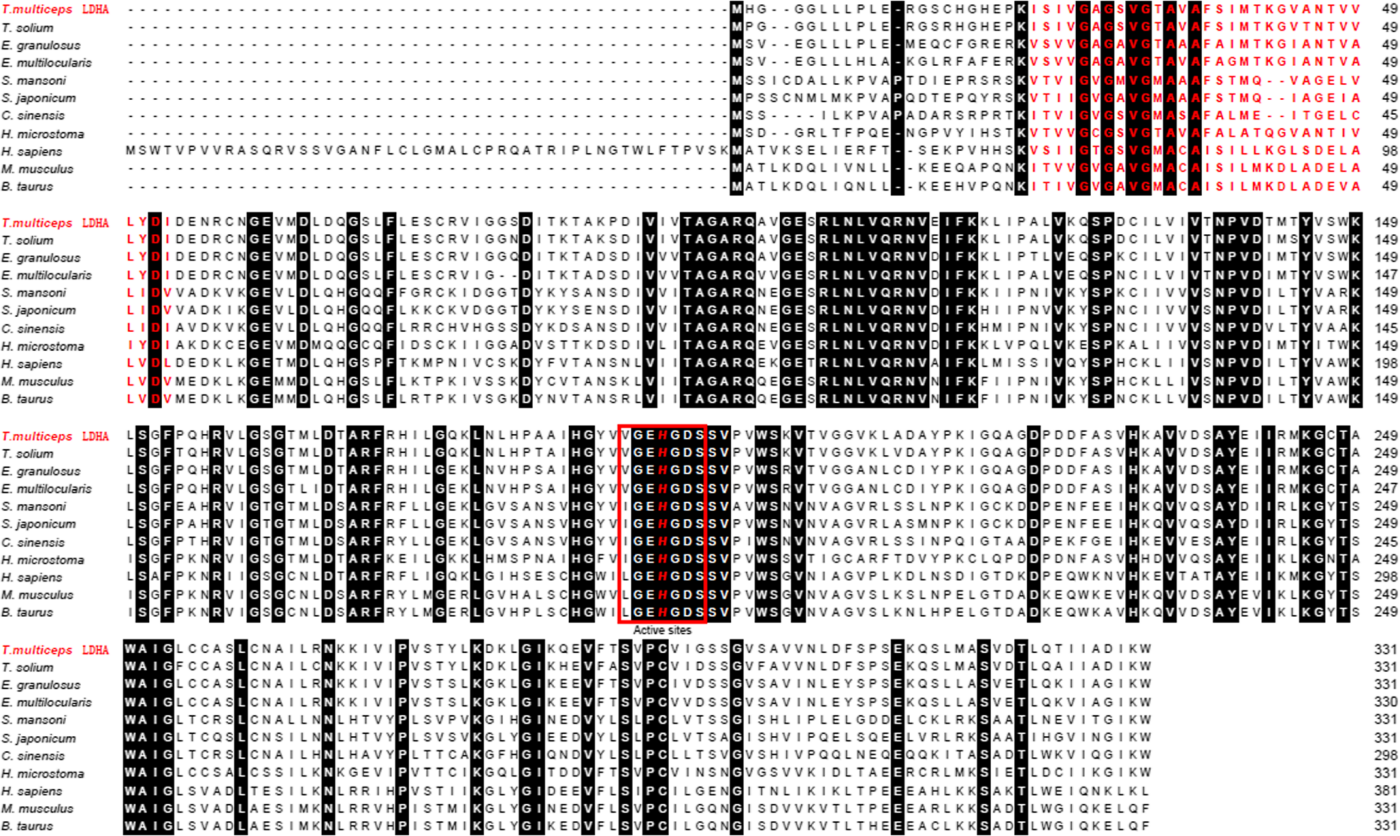

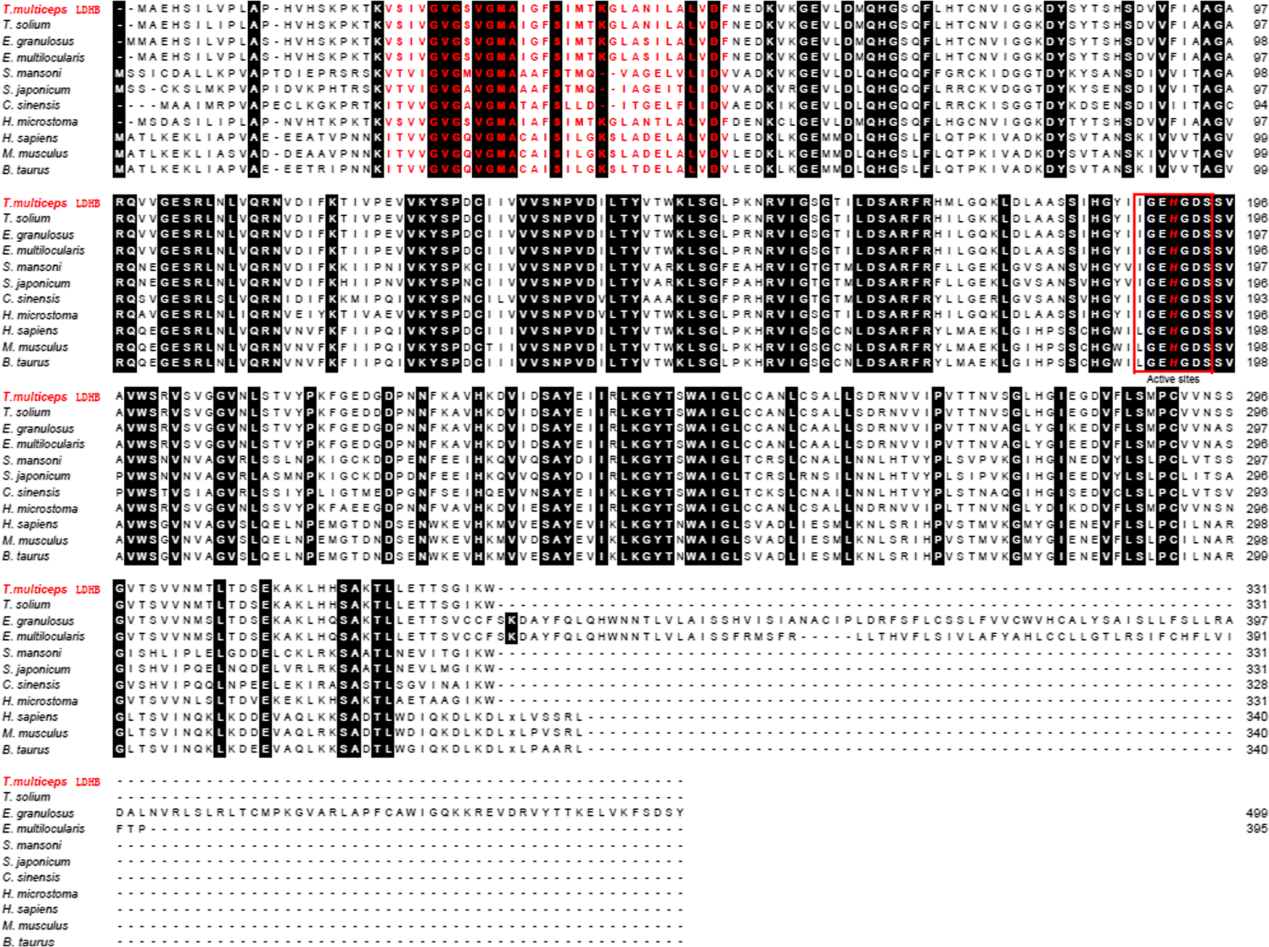
Multiple sequence alignment of LDHA and LDHB in *T. multiceps* and other species. Conserved sites between LDH sequences are shown as *black columns*. The NAD binding sites are marked with *red letters*. The LDH active sites are marked with a *red box*

**Fig. 4:**
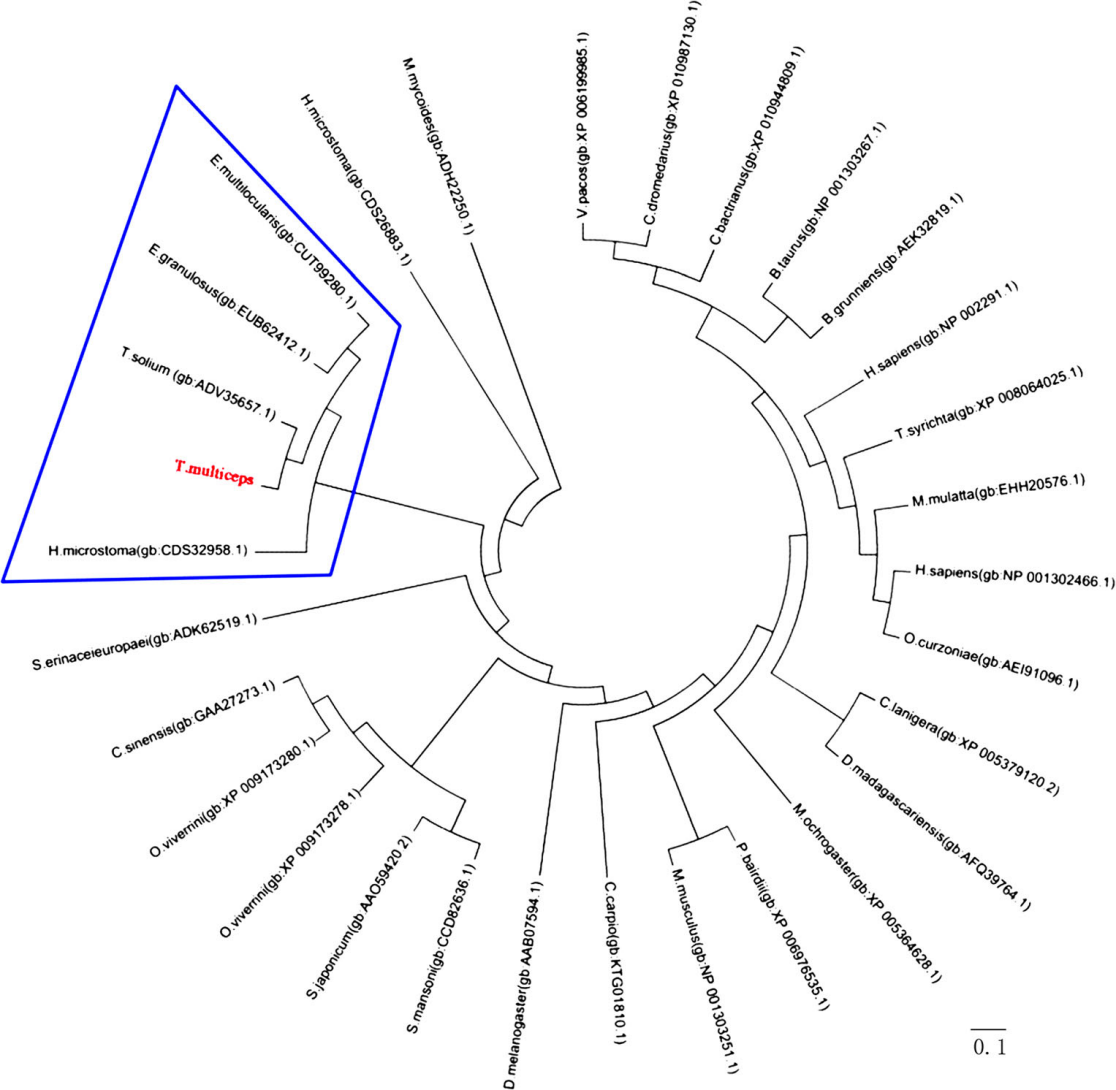
Phylogenetic analysis of LDHB proteins

LDH amino acid sequences from seven different parasite species were retrieved from GenBank, including *T. solium* (GenBank: ADV35656.1/ADV35657.1), *E. granulosus* (GenBank: AFA35122.1/EUB62412.1), *E. multilocularis* (GenBank: CUT99509.1/CUT99280.1), *S. mansoni* (GenBank: CCD82636.1), *S. japonicum* (GenBank: CAX70604.1/AAO59420.2), *C. sinensis* (GenBank: AAV80238.1/GAA27273.1), and *H. microstoma* (GenBank: CDS26883.1/CDS32958.1). Multiple sequence alignment indicated that Tm-LDHA showed 94.56% similarity to Ts-LDHA and Tm-LDHB showed 98.79% similarity to Ts-LDHB (Fig. 3). Based on the sequence alignment, a phylogenetic (neighbor-joining) tree was constructed using the Tm-LDHB sequence and previously published LDHB sequences (Fig. 4). LDHB of *T*. *multiceps* has a relatively close relationship with that of the congeneric cestode *T. solium*, and relatively distant relationships with those from the trematode species *S. japonicum* and *C. sinensis*.

### Expression, purification and identification of recombinant Tm-LDH

**Fig. 5:**
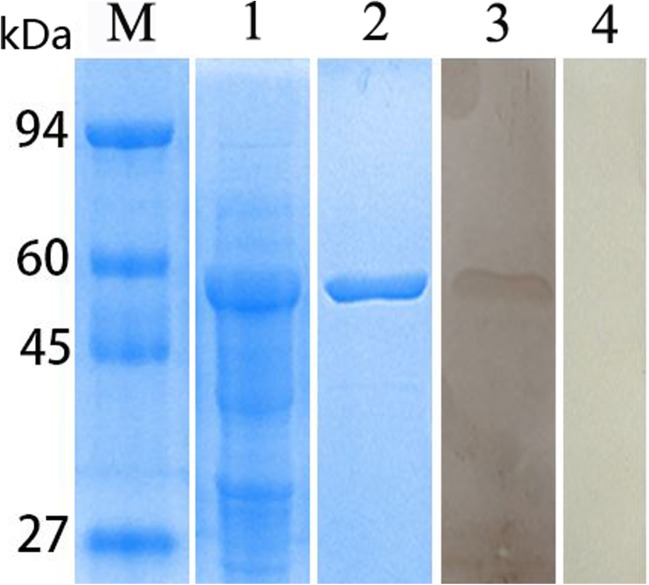
Purification of recombinant *T. multiceps* LDHB and western blot analysis. Lane M: Protein molecular weight markers; 1: Crude extracts of *E. coli* expressing pET32a(+)-Tm-LDH induced by IPTG; 2: Purified recombinant Tm-LDHB; 3: western blot of rTm-LDH incubated with *Coenurus cerebralis*-infected goat serum; 4: western blot of rTm-LDH incubated with negative goat serum

Recombinant Tm-LDHB was expressed with a His-tag. The fusion protein (produced after IPTG induction of *E. coli* for 6 h) showed a single band of 54 kDa on 15% SDS-PAGE (including the His-tag) (Fig. 5, lane 1). rTm-LDHB was primarily present in inclusion bodies. After purification with a Ni-NTA affinity column, rTm-LDHB was subjected to reaction with *Coenurus cerebralis*-infected goat serum. A single band of 54 kDa was observed on the NC membrane (Fig. 5, lane 3), while no band was observed in the negative control (Fig. 5, lane 4), suggesting that rTm-LDHB had good immunoreactivity.

### Establishment, specificity and sensitivity of rTm-LDHB iELISA

After optimization of ELISA conditions, the optimal antigen concentration and serum dilution were determined to be 1.2 μg/well and 1:80, respectively (Table 1). In these conditions, 24 negative serum samples were tested and the cut-off value was calculated as 0.523 (mean + 3SD; mean = 0.4377, SD = 0.0284; data not shown). Therefore, serum samples with OD450 ≥ 0.523 were defined as positive, otherwise they were considered negative. iELISA specificity of 71.42% (10/14) was observed in this study (cross-reactivity with two *E. granulosus*-positive sheep serum samples (*n* = 7) and two *C. tenuicollis*-positive goat serum samples (*n* = 7)). Results of sensitivity testing revealed that the minimum detection limit for positive serum was 1:3200 (mean absorbance = 0. 582).

**Table 1:** Determination of the optimal antigen concentration and serum dilution for indirect ELISA

Antisera at different dilutions		LDH concentration (μg/well)					
		9.6	4.8	2.4	1.2	0.6	0.3
1:20	P	1.747	1.577	1.572	1.485	1.428	1.54
	N	1.301	1.127	1.008	0.924	0.905	0.869
	P/N	1.3428	1.3993	1.5595	1.6071	1.5779	1.7722
1:40	P	1.591	1.378	1.404	1.334	1.225	1.139
	N	1.173	0.976	0.907	0.729	0.639	0.612
	P/N	1.3564	1.4119	1.5480	1.8299	1.9171	1.8611
1:80	P	1.298	1.157	1.223	1.156	0.964	0.971
	N	0.974	0.895	0.814	0.591	0.584	0.566
	P/N	1.3326	1.2927	1.5025	1.9560	1.6507	1.7155
1:160	P	1.121	0.943	0.963	0.864	0.728	0.721
	N	0.864	0.711	0.686	0.531	0.482	0.418
	P/N	1.2975	1.3263	1.4038	1.6271	1.5104	1.7249
1:320	P	0.810	0.710	0.659	0.663	0.490	0.515
	N	0.707	0.559	0.548	0.415	0.363	0.388
	P/N	1.1457	1.2701	1.2026	1.5976	1.3499	1.3273
1:640	P	0.524	0.448	0.44	0.387	0.363	0.335
	N	0.412	0.336	0.278	0.302	0.24	0.223
	P/N	1.2718	1.3333	1.5827	1.2815	1.5125	1.5022

*P* positive serum, *N* negative serum

## Discussion

In parasites, LDH can catalyze reversible reactions between pyruvic acid and lactic acid with the concomitant oxidation of NADH to NAD^+^ (Cook et al. 2014), thus helping parasites use energy (Ramljak et al. 2015). If LDH is inhibited, the development and growth of parasites will cease or they may die (Veerakumari and Munuswamy 2000). In previous studies, researchers mainly focused on the molecular characteristics of LDH and its value for immunological diagnosis and drug development (Dai et al. 2010; Hu et al. 2007). In this study, two full-length cDNAs that encode LDH A (Tm-LDHA) and B (Tm-LDHB) from *T. multiceps* were identified. After amino acid searches against the NCBI database, the highest similarity (94.56–98.79%) was found between Tm*-*LDH and *T. solium* (Ts-)LDH. Simultaneously, these two amino acid sequences show high identities and similar structural characteristics to previously reported LDHA or LDHB sequences from other species.

Study of *E*. *granulosus* LDH revealed that the linear B-cell epitopes aa 101–107, 191–196, and 307–313 related to the function of the LDH, and that LDH was a potential anti-*E. granulosus* drug target (Gan et al. 2012). Further, Du et al. (2011) analyzed the linear B-cell epitopes of TsLDH-A and TsLDH-B and discovered that cestodes have a common, specific epitope, which means LDH is a potential antiparasitic drug target (Du et al. 2011). Using topology analysis, the present study found both Tm-LDHA and Tm-LDHB contained a linear B-cell epitope (aa 191–197, EHGDSSV), which overlaps with the LDH active site (aa 190–195). Given that a key catalytic histidine residue (His192) is located in this potential B-cell antigen epitope, if a specific antibody bound to His192, it could inhibit the function of the enzyme, which may lead to impaired glucose metabolism. Therefore, TmLDH may be an important drug target, consistent with the findings of previous studies (Du et al. 2010a, b). In addition, parasite LDHs can transport lactic acid out of the cell, cause antibody-dependent cell-mediated cytotoxicity, and mediate complement function, so LDH is also a promising vaccine target (Hu et al. 2007).

LDH belongs to an isozyme family. In mammals, LDH is a tetramer composed of three types of subunits, each encoded by a distinct gene (LDH-A, LDH-B and LDH-C). Various tetrameric LDHs are expressed in different tissues. The LDH-B gene is expressed in aerobic tissues, such as the heart, while the LDH-A gene is expressed in lactate-producing tissues, such as skeletal muscle (Alcazar et al. 2000; Imagawa et al. 2006). In this study, subcellular localization showed that Tm-LDHA located in the cytoplasm while Tm-LDHB located in mitochondria; this finding is different from that for the LDHs of *T. solium* (Du et al. 2011).

Early diagnosis is premise of cerebral coenurosis treatment. The autopsy can accurately confirm hydatid infections in goats; however, it is more applicable to later stage and does not meet the need for cerebral coenurosis treatment. Although there are some difficulties such as existence of multiple infections with different taeniid species, antigen cross-reactivity, and low level of specific antibody responses to infection in ungulates infected with taeniid cestodes, it is still necessary to search for highly sensitivety diagnostic antigens (McManus 2014). Encouragingly, some diagnostic recombinant antigens with a high sensitivity such as Tm-GP50, Tm-HSP70, Tm-P2, and Eg-Grx1 have been recently reported in taeniid species (Huang et al. 2016; Huang et al. 2014; Wang et al. 2015; Song et al. 2016). Thereby, it is important to select and assess recombinant antigens with a high sensitivity for diagnosing *C. cerebralis* infection. Previous studies showed that LDH from different parasites, including *T. solium*, *S. japonicum*, *C. sinensis*, and *P. knowlesi*, has potential as a diagnostic antigen (Lv et al. 2007; Huang et al. 2010; Singh et al. 2012; Xiao et al. 1999). In this study, immunoblotting results indicated that goats infected with *T. multiceps* produced specific antibody against Tm-LDHB. Previously, the diagnosis of cerebral coenurosis mainly used cystic fluid and scolex as the diagnostic antigens (Daoud and Herbert 1982; Fu et al. 1997). However, these antigens are difficult to obtain and cannot be commercialized. The recombinant antigens have good specificity, a stable source, and a high diagnostic odds ratio (Mohammadzadeh et al. 2012). Some recombinant diagnostic antigens have been reported for *T. multiceps*, including Tm7, TmHSP70, TmP2, and TmGP50 (Du et al. 2010a, b; Huang et al. 2016, 2014; Wang et al. 2015; An et al. 2011). In this study, we found that the predicted total B-cell epitopes of Tm-LDHB were more than that of Tm-LDHA and established an iELISA method for preliminary evaluation of the serodiagnostic potential of rTm-LDHB. The iELISA exhibited good specificity (71.42%). However, the specificity of rTm-LDHB was lower than that of rTm-P2 (96.3%), rTm-HSP70 (83.3%), and rTm-GP50 (92.6%) (Huang et al. 2015; Wang et al. 2015; Huang et al. 2016); moreover, we observed cross-reactivity with *E. granulosus*-positive sheep sera and *C. tenuicollis*-positive goat sera. These results showed that rTm-LDH-B is not a specific antigen candidate for immunodiagnosis.

## References

[CR1] Alcazar O, Tiedge M, Lenzen S (2000). Importance of lactate dehydrogenase for the regulation of glycolytic flux and insulin secretion in insulin-producing cells. Biochem J.

[CR2] Al-Riyami S, Ioannidou E, Koehler AV, Hussain MH, Al-Rawahi AH, Giadinis ND, Lafi SQ, Papadopoulos E, Jabbar A (2015). Genetic characterisation of *Taenia multiceps* cysts from ruminants in Greece. Infect Genet Evol.

[CR3] An XX, Yang GY, Wang YW, Mu J, Yang AG, Gu XB, Yang YD, Wei LF, Wen JG, Wang SX, Bian R (2011). Prokaryotic expression of Tm7 gene of *Taenia multiceps* and establishment of indirect ELISA using the expressed protein. Act Vet Zootechn Sin.

[CR4] Chen ZY, Dai JL, Huang J, Shen PX, Liao XJ (2010). Effect of praziquantel, albendazole and mebendazole on recombinant lactate dehydrogenase of *Taenia asiatica*. Chin J Public Health.

[CR5] Cook WJ, Senkovich O, Hernandez A, Speed H, Chattopadhyay D (2014). Biochemical and structural characterization of *Cryptosporidium parvum* lactate dehydrogenase. Int J Biol Macromol.

[CR6] Crowther JR (2009) The Elisa Guidebook: Second Edition. Humana Press, NewYork, USA

[CR7] Dai JL, Huang J, Li B, Liao XJ, Wang Y (2010). Prokaryotic expression of lactate dehydrogenase from *Teania saginata* and its immunological identification. Chin J Public Health.

[CR8] Dando C, Schroeder ER, Hunsaker LA, Deck LM, Royer RE, Zhou X, Parmley SF, Vander Jagt DL (2001). The kinetic properties and sensitivities to inhibitors of lactate dehydrogenases (LDH1 and LDH2) from *Toxoplasma gondii*: comparisons with pLDH from *Plasmodium falciparum*. Mol Biochem Parasitol.

[CR9] Daoud IS, Herbert IV (1982). Isolation of two lipoprotein antigens from the metacestodes of *Taenia hydatigena* (Pallas, 1766) and *Taenia multiceps* (Leske, 1780) and their evaluation in sero-diagnosis. Vet Parasitol.

[CR10] Dehghani M, Mohammadi MA, Rostami S, Shamsaddini S, Mirbadie SR, Harandi MF (2016). High-resolution melting analysis (HRM) for differentiation of four major Taeniidae species in dogs *Taenia hydatigena*, *Taenia multiceps*, *Taenia ovis*, and *Echinococcus granulosus* sensu stricto. Parasitol Res.

[CR11] Du WY, Dai JL, Huang Y, Hu XC, Yu XB, Xu J, Liao XJ (2010). Bioinformatics analysis and comparison of the genes encoding lactate dehydrogenase A and B from *Taenia solium*. J Trop Med.

[CR12] Du WY, Huang J, Hu XC, Yu XB, Xu J, Liao XJ, Dai JL (2010). Sequence analysis, cloning expression and immunogenicity analysis of lactate dehydrogenase gene from *Taenia solium*. Chin J Zoonoses Chin J.

[CR13] Du WY, Hu FY, Yang YB, Hu D, Hu XC, Yu XB, Xu J, Dai JL, Liao XJ, Huang J (2011). Molecular cloning, characterization, and immunolocalization of two lactate dehydrogenase homologous genes from *Taenia solium*. Parasitol Res.

[CR14] Fu B, Dou L, Chai Z, Zhu X, Sun X (1997). Studies on the antigens of muticeps cestode—analysis of muticeps adult antigens and the excretory-secretory antigens of *Coenurus cerebralis* protoscolex with SDS-PAGE. Chin J Vet Parasitol.

[CR15] Gan W, Zhang ZP, Lv G, Xu HX, Zeng SX, Li YZ, Wu WP, Hu XC (2012). The topological structure and function of *Echinococcus granulosus* lactate dehydrogenase, a tegumental transmembrane protein. Mol Biochem Parasitol.

[CR16] Hu XC, Xu J, Lv G, Huang C, Yu XB (2007). Bioinformatics analyze the structure and characteristics of the gene and protein of *Clonorchis sinensis* lactate dehydrogenase. J Trop Med.

[CR17] Huang C, Wang LX, Hu XC, Yu XB, Huang Y, Deng CH, Xu J (2010). Clone, expression, and characterization of two epitopes E10-20 and E94-102 of lactate dehydrogenase from *Clonorchis sinensis*. J Sun Yat-Sen Univ (Med Sci).

[CR18] Huang Y, Yi DY, Liu LL, Huang L, Yu WJ, Wang Q, Li YQ, Han XM, Qiu DC, Wang N, Wu WP, Health DD (2014). Echinococcus infections in Chinese dogs: a comparison of coproantigen kits. J Helminthol.

[CR19] Huang X, Chen L, Yang YD, Gu XB, Wang Y, Lai WM, Peng XR, Yang GY (2015). Expression, tissue localization and serodiagnostic potential of *Taenia multiceps* acidic ribosomal protein P2. Parasit Vectors.

[CR20] Huang X, Xu J, Wang Y, Guo C, Chen L, Gu XB, Lai WM, Peng XR, Yang GY (2016). GP50 as a promising early diagnostic antigen for *Taenia multiceps* infection in goats by indirect ELISA. Parasit Vectors.

[CR21] Imagawa T, Yamamoto E, Sawada M, Okamoto M, Uehara M (2006). Expression of lactate dehydrogenase-A and -B messenger ribonucleic acids in chick glycogen body. Poult Sci.

[CR22] Li WH, Qu ZG, Zhang NZ, Yue L, Jia WZ, Luo JX, Yin H, Fu BQ (2015). Molecular characterization of enolase gene from *Taenia multiceps*. Res Vet Sci.

[CR23] Liwak U, Ananvoranich S (2009). *Toxoplasma gondii*: over-expression of lactate dehydrogenase enhances differentiation under alkaline conditions. Exp Parasitol.

[CR24] Lu Y, Jia R, Wang M, Xu Y, Zhu D, Chen S, Liu M, Yin Z, Chen X, Cheng A (2014). In vitro expression and development of indirect ELISA for capsid protein of duck circovirus without nuclear localization signal. Int J Clin Exp Pathol.

[CR25] Lv G, Hu XC, Huang C, Li YW, Xu J, Wu ZD, Yu XB (2007). Prokaryotic expression, purification and identification of lactate dehydrogenase from *Schistosome japonicum*. Chin J Public Health.

[CR26] McManus DP (2014). Immunodiagnosis of sheep infections with *Echinococcus granulosus*: in 35 years where have we come?. Parasite Immunol.

[CR27] Merbl Y, Shilo-Benjamini Y, Chai O, Chamisha Y, Anglister N, King R, Horowitz I, Aizenberg Z, Shamir MH (2014). *Taenia multiceps* brain cyst removal in two wild Nubian ibex (*Capra nubianas*). J Zoo Wildl Med.

[CR28] Mohammadzadeh T, Sako Y, Sadjjadi SM, Sarkari B, Ito A (2012). Comparison of the usefulness of hydatid cyst fluid, native antigen B and recombinant antigen B8/1 for serological diagnosis of cystic echinococcosis. Trans R Soc Trop Med Hyg.

[CR29] Nie HM, Xie Y, Fu Y, Yang YD, Gu XB, Wang SX, Peng X, Lai WM, Peng XR, Yang GY (2013). Cloning and characterization of the fatty acid-binding protein gene from the protoscolex of *Taenia multiceps*. Parasitol Res.

[CR30] Oryan A, Amrabadi O, Sharifiyazdi H, Moazeni M, Akbari M, Ghane M (2015). Application of polymerase chain reaction on cerebrospinal fluid for diagnosis of cerebral coenurosis in small ruminants. Parasitol Res.

[CR31] Oryan A, Moazeni M, Amrabadi O, Akbari M, Sharifiyazdi H (2015). Comparison of distribution pattern, pathogenesis and molecular characteristics of larval stages of *Taenia multiceps* in sheep and goats. Small Rumin Res.

[CR32] Ramljak S, Schmitz M, Zafar S, Wrede A, Schenkel S, Asif AR, Carimalo J, Doeppner TR, Schulz-Schaeffer W, Weise J (2015). Cellular prion protein directly interacts with and enhances lactate dehydrogenase expression under hypoxic conditions. Exp Neurol.

[CR33] Singh V, Kaushal DC, Rathaur S, Kumar N, Kaushal NA (2012). Cloning, overexpression, purification and characterization of *Plasmodium knowlesi* lactate dehydrogenase. Protein Expr Purif.

[CR34] Song XJ, Yan M, Hu DD, Wang Y, Wang N, Gu XB, Peng XR, Yang GY (2016). Molecular characterization and serodiagnostic potential of a novel dithiol glutaredoxin 1 from *Echinococcus granulosus*. Parasit Vectors.

[CR35] Varcasia A, Tamponi C, Tosciri G, Pipia AP, Dore F, Schuster RK, Kandil OM, Manunta ML, Scala A (2015). Is the red fox (*Vulpes vulpes*) a competent definitive host for *Taenia multiceps*?. Parasit Vectors.

[CR36] Veerakumari L, Munuswamy N (2000). In vitro effect of some anthelmintics on lactate dehydrogenase activity of *Cotylophoron cotylophorum* (Digenea: paramphistomidae). Vet Parasitol.

[CR37] Wang Y, Nie H, Wang T, Huang X, Chen L, Lai W, Peng X, Yang G (2015). An ELISA using recombinant TmHSP70 for the diagnosis of *Taenia multiceps* infections in goats. Vet Parasitol.

[CR38] Xiao SH, You JQ, Guo HF, Mei JY, Jiao PY, Yao MY, Zhuang ZN, Feng Z (1999). Effect of artemether on phosphorylase, lactate dehydrogenase, adenosine triphosphatase, and glucosephosphate dehydrogenase of *Schistosoma japonicum* harbored in mice. Acta Pharmacol Sin.

[CR39] Yang G, Jing CX, Zhu PX, Hu XC, Xu J, Wu ZD, Yu XB (2006). Molecular cloning and characterization of a novel lactate dehydrogenase gene from *Clonorchis sinensis*. Parasitol Res.

